# Current News and Opinion

**Published:** 1884-04

**Authors:** 


### Current News and Opinion.

### ANOTHER NATIONAL DENTAL SOCIETY.

*To the President and Members of the State Dental Associations:*

GENTLEMEN: The foremost men of our profession admit the fact that the name of American Dentistry has not that world-wide recognition which its honor demands and its history would warrant. In our opinion, this is an opportune time for a general movement to place it upon a basis commensurate with its dignity, and we venture to submit the outline of a plan for the purpose, which we think feasible, and which we hope will meet with your sanction and furtherance.

As a nation claiming to lead the world in the science and art of dentistry, we should have one great repesentative body of the profession to speak forth with authority its aims, duties and attainments.

Let us, therefore, organize a National Dental Association of the United States, composed of delegates elected by the various State organizations, an equal number from each State, to meet annually, and always at Washington, D. C. Thus, we would give to Delaware the same voice as to New York. In order always to secure a full meeting, we would suggest six as the number of delegates from each State; a like number of alternates having been elected. Let the National Association elect a Board of Regents, one from each State, who shall control its meetings, setting the time, etc.

As important adjuncts to this Association, we believe it would be found expedient to establish an extensive library, to contain dental works and publications, standard and periodical, foreign and native; and the founding of a national museum, to illustrate the past, present and future of dentistry.

The wonderful benefit to the profession and humanity in general to be derived from these is certainly manifest to every intelligent dentist.

We are sure that room can be obtained in the Smithsonian Institute for the museum, and in the library of the Surgeon General's office for the library. We are equally sure that Congress will grant an appropriation for both purposes, as is done for the medical profession.

Mature deliberation at the meeting for organizing will suggest the details of a scheme, which we have not thought it necessary to go into here.

In conclusion, we would respectfully urge that you elect, at your present meeting, delegates, with alternates, to meet for the purpose of organization, at the time agreed upon by the chairman of the different State delegations.

Do this in anticipation of like action upon the part of other State societies; then forward to Dr. B. H. Catching, Atlanta, Ga., the name and address of your chairman, and he will act as medium of communication between the different delegations, informing each of the action of the other, thereby bringing about unity of action.

Yours respectfully,

FRANK ABBOTT, New York City.
J. E. CRAVENS, Indianapolis, Ind.
C. T. STOCKWELL, Springfield, Mass.
F. SEARLE, Springfield, Mass.
W. C. WARDLAW, Augusta Ga.
B. H. CATCHING, Atlanta, Ga.

---

### COLLEGE COMMENCEMENTS.

The Eighteenth Annual Commencement Exercises of the New York College of Dentistry were held at Chickering Hall, New York, on Thursday evening, March 6th, 1884.

GRADUATES.

| | | | |
|---|---|---|---|
| John J. R. Beer, | Switzerland. | Manuel B. Lèmus, | U. S. Columbia. |
| Henry A. Benedict, | N. J. | Roderick M. Lowe, | N. Y. |
| Cornelius E. Byrne, | N. Y. | William Lowenthal, | N. J. |
| Charles L. Berger, | N. Y. | Charles W. Le Beau, | Ohio. |
| Charles F. Burley, | Conn. | William H. Milke, | Pa. |
| Arthur J. Blersch, | Germany. | Edward T. Mason, | N. Y. |
| William J. Caillè, Jr., | N. Y. | Geo. G. Neish, | Canada. |
| Luis M. Clement, | Panama. | Edwin F. Normand, | N. Y. |
| Oliver L. Cole, | N. Y. | James J. Pruden, | N. Y. |
| Edgar M. Davis, | N. Y. | Walter E. Parrott, | N. Y. |
| Francis Eschauzier, | Spain. | Eugenio E. Quesada, | Cuba. |

| | | | |
|---|---|---|---|
| Raymund Faquinetto, | Cuba. | José B. Ross, | Conn. |
| Edmond A. Ferguson, | N. Y. | James A. Roberts, | N. H. |
| Charles B. Gates, | N. Y. | Edward J. Ranhoffer, | N. Y. |
| Friedrich Grau, | Germany. | Joseph J. Strohmeyer, | N. Y. |
| Edward L. Goodell, | N. Y. | Nathan Sanders, | N. Y. |
| Stephen S. Hawley, | La. | William M. Stanbrough, | N. Y. |
| Arthur C. Hull, | N. H. | James W. Taylor, | Uruguay, S. A. |
| Herman G. Hichborn, | Me. | Charles T. Van Derlip, | N. Y. |
| Eugene L. Johnson, | N. Y. | Wesley Wait, | N. Y. |
| William R. Libby, | N. Y. | Karl R. O. Wendler, | Germany. |

Number of matriculants, 142.

---

The Twenty-eighth Annual Commencement Exercises of the Pennsylvania College of Dental Surgery were held at the American Academy of Music, Philadelphia, Thursday, February 28th, 1884.

GRADUATES.

| | | | |
|---|---|---|---|
| Laurence C. Anderson, | Miss. | William Koehncke, | Germany. |
| George S. Andrews, | N. Y. | Prosper A. Ladmore, L. D. S., | Eng. |
| Thomas Ashton, | Pa. | Hugh J. Laird, | Pa. |
| William L. Bacon, | Va. | Henry Leffman, M. D., | Pa. |
| Maria Katherine Benkard, | Germany. | Alex. P. Long, | Pa. |
| E. R. C. Blackburn, | Pa. | Victor Magliocco, | Pa. |
| C. Curtis Coffee, | Ohio. | Henry Maser, | Ohio. |
| George W. Cupit, | Pa. | C. H. M. Neall, | Pa. |
| George Culbertson, | Pa. | Nazario Noguera, | South America. |
| Maria D. C. Dalmer, | Germany. | Enrique Noguara, | South America. |
| Vasco A. Da Silva, | N. Y. | Harvey E. Nyer, | Pa. |
| Fremont D. Davis, | Ohio. | Juan Ph. Orozco, | Salvador, C. A. |
| Heinrich Deutschmann, | Germany. | Pedre A. Palacio, | Cuba. |
| William P. Dickinson, | Iowa. | Joseph A. Phillips. | Pa. |
| J. Frank Dougherty, | Ohio. | Paula Pratz, | Germany. |
| Sophie E. Feltwell, | Pa. | Charlotte Renard, | Germany. |
| Alexander L. Foerster, | Pa. | Guy C. Rich, | N. Y. |
| August Gassner, | Germany. | Nicholas Rocha, | U. S. of Col., S. A. |
| A. M. Green, | Pa. | J. Sabater Rivera, | Porto Rico. |
| Hermann Gutzwiller, | Switzerland. | John Schembs, | Pa. |
| A. W. Hafer, | Pa. | Valentine Schmitt, | Germany. |
| James J. Hamilton, | Pa. | Courtland S. Service, | Pa. |
| Louis Haubeil, | Germany. | Marcus Sichel, | Germany. |
| Ferdinand Hegemann, | Germany. | T. Frank Spencer, | R. I. |

| | | | |
|---|---|---|---|
| Edwin H. Hewit, . . . . . | Wis. | Herman Stocker, . | Switzerland. |
| Kurtz P. Hill, . . . . . | Pa. | Josè S. Tamayo, | U. S. of Col., S. A. |
| Pantaleon Hoyos, | U. S. of Col., S. A. | Robert Addison Todd, . | Montana. |
| Parker B. Hunt, . . . . | Pa. | O. S. Watrous, . . . . | Conn. |
| G. L. S. Jàmeson, . . . . | Canada. | Charles Wetzel, . . . . | W. Va. |
| George A. Jarvis, . . . | Mich. | Frederike Wiesner, . . . | Austria. |
| George Norman Johnson, . | N. H. | Marvin A. Wint, . . . . | Pa. |
| Arthur S. Kniffin, . . . . | N. J. | William N. Wirt, . . . . | Ind. |

Number of matriculants, 140.

---

### LEGISLATION.

The following is a copy of the amendment to the Dental Law of the State of New Jersey, which has passed both branches of the Legislature. It originated with the very efficient committee appointed for the purpose, and was taken in charge by Drs. Brown and Clarke, who have persistently pushed it to a successful issue:

1. Be it enacted *by the Senate and General Assembly of the State of New Jersey,* That the first section of the act to which this is a supplement, shall be amended so as to read as follows:

[Be it enacted *by the Senate and General Assembly of the State of New Jersey,* That from and after the passage of this act it shall be unlawful for any person not now lawfully practicing to engage in the practice of denistry in the State of New Jersey unless said person has graduated and received a diploma from the faculty of a reputable dental college chartered under the authority of some one of the United States, and that any person hereafter engaging in the practice of dentistry in the State shall within one month after commencing such practice register his name in a book, kept for that purpose in the county clerk's office of the county in which he shall have engaged in the practice of dentistry, giving his name and the name of the dental college of which he is a graduate, and the name of the place in which he shall have engaged in practice, and for which registry the said county clerk shall be entitled to demand and receive from each person registering the sum of fifty cents; and any person violating any of the provisions of this act shall be liable to the penalties prescribed in the sixth section of the act to which this is a supplement.]

Fred. A. Levy,
Chas. A. Meeker,
James C. Clarke,
James G. Palmer,
George C. Brown,
*Committee.*

### A SIMPLE OPERATION FOR FACIAL NEURALGIA.

Dr. J. F. Heustis, of Alabama, describes a simple operation for the relief of tic douloureux (*Med. News*, Dec. 8th), which is worthy of further trial. Discarding Carnochan's operation of trephining the antrum and following up the nerve beneath the orbit and removing it, and Langenbeck's slighter one of dividing the nerve far back in the orbit with a tenotome and drawing it out through the infra-orbital foramen, Dr. Huestis cut down upon the infra-orbital foramen, and with a fine steel drill, such as dentists use, improvised of piano-wire, dilled out the nerve in its entire length, as far back as the spheno-maxillary fissure. The immediate effect of this operation was to abolish all sensation in the previous by sensitive parts, and to enable the patient to use the jaws without suffering the darting pains formerly experienced.—*Maryland Medical Journal.*

### REGISTERING VOCAL SOUNDS.

Professor Harrison Allen has, according to the *Philadelphia Record*, invented an instrument by which spoken language can be represented by a series of curved lines on a receiving surface. These experiments originated from observations on the soft palate, and he is now able to register the various phonetic sounds of the human voice. Prof. Allen has already shown by the aid of this device that many of the sounds which have long been considered by elocutionists to be formed by the direct action of the lips, the teeth, or the tongue, are in reality formed by the action of the palate. The subject is sure to prove of interest to the world of science, and Prof. Allen thinks it may develop some very interesting facts.

### AN ERROR.

We were mistaken when, in the March number, we said that Dr. E. A. Bogue had accepted the chair of Operative Dentistry in the *Institut Odontotechnique de France.* He has only consented to act as one of the Clinical Demonstrators. We gave the item of the authority of an English journal.

### "IT PAYS WELL."

Said an esteemed professional associate not long since: "I am glad that you induced me to subscribe for the INDEPENDENT PRACTITIONER, for I like it ever so much. A single suggestion I got from a recent number is worth more to me than I paid for the entire volume."

Another friend, a few days ago, remarked: "Miller's article in the February number of the INDEPENDENT PRACTITIONER, on the causes of caries, is an exceedingly valuable paper, and I read it with great interest. This alone paid me well for subscribing."

Members of our calling progressively inclined, and who are desirous of keeping pace with the advance which every month discloses, will find that it "pays well" to become familiar with the contents of our dental periodicals. Try it. C. E. F.

---

### CENTRAL DENTAL ASSOCIATION OF NORTHERN NEW JERSEY.

At the annual election of the above named Society, held February 18th, at the office of Drs. Richards and Levy, Orange, N. J., the following members were chosen:

President—Fred'k C. Barlow, Jersey City; Vice-President, G. Carleton Brown, Elizabeth; Secretary, B. F. Luckey, Paterson; Treasurer, Chas. A. Meeker, Newark; Executive Committee, S. C. G. Watkins, Montclair; W. P. Richards, Orange; James G. Palmer, New Brunswick; R. M. Sanger, Brick Church; G. C. Brown, Elizabeth.

B. F. LUCKEY, D. D. S., Secretary.

---

### DOCTOR LORD'S "SICKLES."

The number of inquiries received concerning these valuable instruments, alluded to in the December number of the INDEPENDENT PRACTITIONER, seems to call for a general reply. They were *devised* by Dr. Lord of New York, but *not* "patented," as it is contrary to the nature of this gentleman to secure an individual grasp upon anything he can contribute to benefit his fellows. The "sickles," of various sizes, can be procured at the dental depots. C. E. F.

### DENTAL SOCIETY MEETINGS FOR APRIL.

Alabama Dental Association.—At Birmingham the second Tuesday.

Texas Dental Association.—In San Antonio, the last Tuesday.

Brooklyn Dental Society.—Second Monday evening.

New York Odontological Society.—Third Tuesday evening.

Fifth District Society of the State of New York.—At Utica, April 8th and 9th.

Eighth District Society of the State of New York.—At Buffalo, April 15th and 16th.

---

### SPECIFIC DISEASE IN THE LOWER ANIMALS.

Martineau has succeeded in inoculating a monkey with syphilis, obtaining first a hard chancre, later, specific skin eruptions, and after ten months a syphilitic ulcer on the soft palate.

---

### IODOFORM IN SURGERY.

The deaths in Billroth's clinic in 1881 were 9.5 per cent. In 1882 it sank to 6.3 per cent. Iodoform gets the credit for the improvement.

---

### JOURNALISTIC BIRTHS AND DEATHS.

*The Philadelphia Medical News* says that during the past year fifty-five new medical journals have appeared, and fifteen have died.

---

### REMOVAL.

Alfred J. Nickolds, manufacturer of gold foils, Brooklyn, N. Y., has removed to No. 238 McDonough Street.

